# The Development of a Framework to Support Ageing Well in the Torres Strait and Northern Peninsula Area of Australia

**DOI:** 10.1111/ajag.70211

**Published:** 2026-07-27

**Authors:** Rachel Quigley, Sarah Russell, Edward Strivens, Betty Sagigi, Chenoa Wapau, Sarah Larkins, Sean Taylor, Leon Flicker, Kate Smith, Dina LoGiudice, Nancy A. Pachana, Michelle Redman‐MacLaren

**Affiliations:** ^1^ College of Medicine and Dentistry James Cook University Cairns Queensland Australia; ^2^ Cairns and Hinterland Hospital and Health Service Cairns Queensland Australia; ^3^ Torres and Cape Hospital and Health Service Thursday Island Queensland Australia; ^4^ Melbourne School of Health Sciences University of Melbourne Parkville Victoria Australia; ^5^ Western Australian Centre for Health and Ageing University of Western Australia Crawley Western Australia Australia; ^6^ Boola Boola Djinda Medical School University of Western Australia Perth Western Australia Australia; ^7^ Department of Medicine, Royal Melbourne Hospital University of Melbourne Parkville Victoria Australia; ^8^ School of Psychology The University of Queensland Brisbane Queensland Australia; ^9^ Faculty of Nursing, Medicine and Health Sciences Solomon Islands National University Honiara Solomon Islands

**Keywords:** Aboriginal and Torres Strait Islander Peoples, chronic disease, dementia, healthy ageing, holistic health

## Abstract

**Objective:**

Older Aboriginal and Torres Strait Islander Australians are central to their communities, providing cultural leadership and care. However, colonisation and systemic inequities have led to significant health disparities, with chronic diseases and dementia disproportionately affecting those aged over the age of 55 years. This study aimed to develop a strengths‐based framework to support healthy ageing in the Torres Strait and Northern Peninsula Area.

**Methods:**

A participatory action research approach was conducted across five communities, involving yarning circles with 45 community members, clinical audits of 1128 residents using the Healthy Ageing Audit Tool (HAAT) and continuous quality improvement (CQI) initiatives. Findings informed the co‐design of an Ageing Well Framework, refined through stakeholder workshops and community feedback.

**Results:**

The HAAT audit revealed high rates of chronic disease and multimorbidity among adults aged over 55 years, alongside gaps in preventive care, including low rates of cardiovascular risk and dementia screening and limited follow‐up for abnormal findings. Continuous Quality Improvement (CQI) activities highlighted opportunities to improve culturally appropriate care, such as increased use of Indigenous Health Workers, validated screening tools and comprehensive health assessments. The co‐designed Ageing Well Framework outlined strategies at community, primary health care and individual levels to promote cultural and social connectedness, independence and ageing in place.

**Conclusion:**

The Ageing Well Framework provides a culturally responsive, evidence‐based guide to improving health and well‐being for older Aboriginal and Torres Strait Islander Peoples. It fosters collaboration across sectors and prioritises cultural determinants of health, supporting holistic care and addressing health inequities in the Torres Strait and Northern Peninsula Area (NPA).

## Practice Impact

The Ageing Well Framework takes a holistic strengths‐based approach to support cultural, physical, cognitive and social functional ability. It provides strategies and recommendations for a whole‐of‐community response to enable older adults to flourish, stay connected and live meaningful lives, and continue living at home and within their communities.

## 1 Introduction

Older Aboriginal and Torres Strait Islander Australians play a crucial role in the health of their communities, including holding cultural rights and responsibilities for maintaining connections to Country, caring for extended family members and providing leadership and support within their families and communities [1].

As with all populations, Aboriginal and Torres Strait Islander Peoples seek to age well by remaining active, healthy and independent for as long as possible. However, the historical and ongoing effects of colonisation and racism have contributed to enduring inequities in the health and well‐being of Aboriginal and Torres Strait Islander Peoples, and disparities in the ageing trajectory [2]. This cumulative disadvantage across the life span, and intergenerationally, negatively affects experiences of ageing well [3]. Long‐term health conditions affect 9 in 10 Aboriginal and Torres Strait Islander Peoples aged 55 years or older, with higher risks of chronic conditions, including diabetes, cardiovascular disease, respiratory disease and dementia [2, 4, 5]. Maintaining the health of older adults is crucial not only for their quality of life but also the cohesiveness of the community they live in.

The development of a framework to support ageing well in the Torres Strait and Northern Peninsula Area (NPA) arose from over two decades of work by the Healthy Ageing Research Team (HART), a multidisciplinary team of Torres Strait Islander, Aboriginal and non‐Indigenous clinicians and researchers with more than 25 years' experience in gerontological service delivery and research with Torres Strait and NPA communities. Their work is grounded in long‐standing partnerships and sustained consultation with local health services, community members and councils [6, 7].

A dementia prevalence study conducted by the team across the Torres Strait identified a prevalence of 14%, nearly three times the national rate, driven by high rates of midlife chronic disease [4, 8]. In response to study findings, community members identified the need for a strengths‐based framework to support ageing well, emphasising that many older adults live long and healthy lives and that understanding these strengths could benefit the broader community.

The aim of this study was to develop a framework to support ageing well in the Torres Strait and NPA. The study involved exploring localised understandings of ageing well and examining how primary health care and the broader community can enable ageing in place and support individuals to maintain meaningful lives within their communities.

## 2 Methods

**FIGURE 1:**
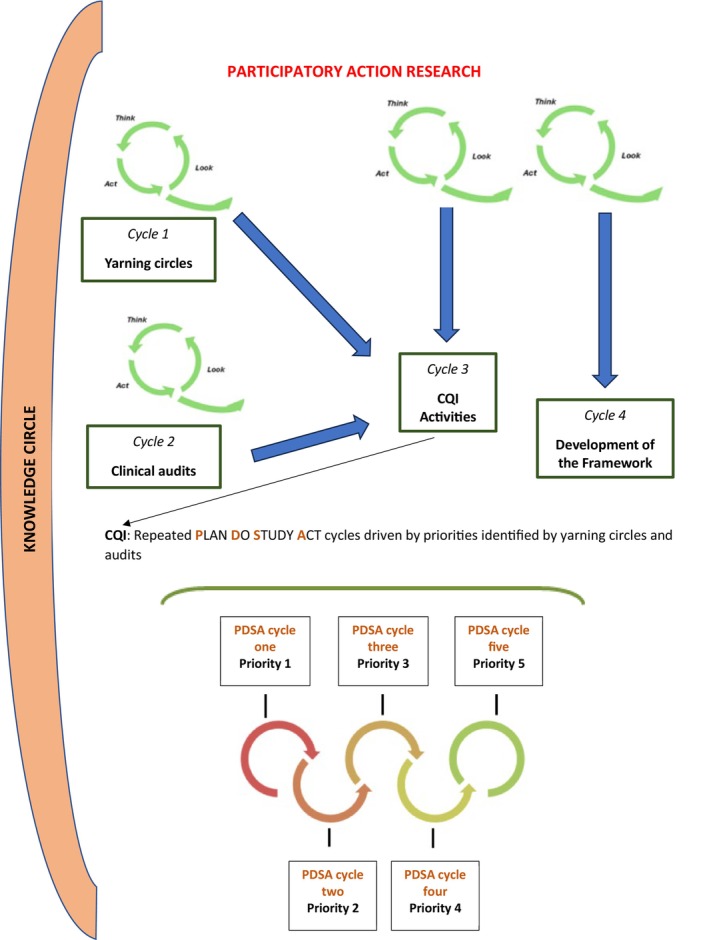
Overview of study design.

A participatory action research (PAR) study was facilitated with five Torres Strait and NPA communities. Figure 1 displays an overview of the study design.

The study comprised four PAR cycles. In Cycle 1, 45 community members participated in 10 yarning circles to explore their understandings of ageing well, including key concepts, enablers and barriers. Findings from this cycle have been reported previously [7].

Cycle 2 involved developing the Healthy Ageing Audit Tool (HAAT) and auditing clinical health service data for residents aged 18 years or older across five Queensland Health Primary Health Care Centres (PHCC) in the region (*n* = 1128). Where applicable, a ≥ 55‐year cut‐off aligned with the age groups in the Aboriginal and Torres Strait Islander Health Assessment, Medicare Benefits Schedule (MBS) item 715 (715HA), which provide a structured approach to preventive care within primary health care. These Indigenous‐specific health assessments provide the opportunity for screening, subsequent care plan development and the delivery of evidence‐based care to support ageing well. Cognitive data were treated separately, with the HAAT auditing assessments for clients aged 45 years or older reflecting evidence of cognitive impairment from this age in the region [4].

Patient record audits provided evidence on the frequency of recommended care [9] and generated clinical service data not captured in existing information systems. The objective was to identify strengths, gaps and limitations in current clinical practice and service delivery to inform PHCC quality improvement activities.

The HAAT was informed by existing best practice guidelines for ageing and chronic disease management [10, 11, 12, 13] and built on previous audits conducted in Indigenous communities focusing on healthy ageing and dementia [14, 15]. The HAAT comprised 73 items capturing clinical performance and health indicators across 12 domains: demographics; attendance at health service (including MBS claims); diagnosis and medications; risk factors, management and review; clinical measurements and investigations; systems examination; scheduled services; mental health and socioemotional well‐being; cognitive functioning; support services (including aged care); Allied Health involvement; and functional assessment.

The HAAT also assessed the Health Service Response (HSR) to any abnormal findings or areas of concern identified through test results, screening or clinical consultations. A HSR included any of the following: repeat testing ± monitoring; prescribing, modifying or deprescribing medication; lifestyle behaviour advice; implementing or reviewing management plans; instigating further investigations; referring onto Allied Health professionals or medical specialists; referring to internal or external programs/interventions; providing information on apps/online resources; prescribing assistive devices; and transferring to hospital for further treatment.

A list of all patients aged 18 years or older was generated from the PHCC general practice software. Eligible patients were those aged 18 or older, residing in the community where the audit was conducted, and having attended the health service for a face‐to‐face or telehealth appointment within the previous 12 months. Electronic medical records of all eligible patients from four of the five participating PHCCs were audited. At the fifth site, a random sample (*n* = 300) was audited due to the large number of eligible clients. Audits were conducted between March 2022 and August 2023 concurrently with PAR Cycle 1. The audit collected data for the 12 months preceding the audit. Descriptive statistics were generated, summarising the sample using frequencies and proportions.

**FIGURE 2:**
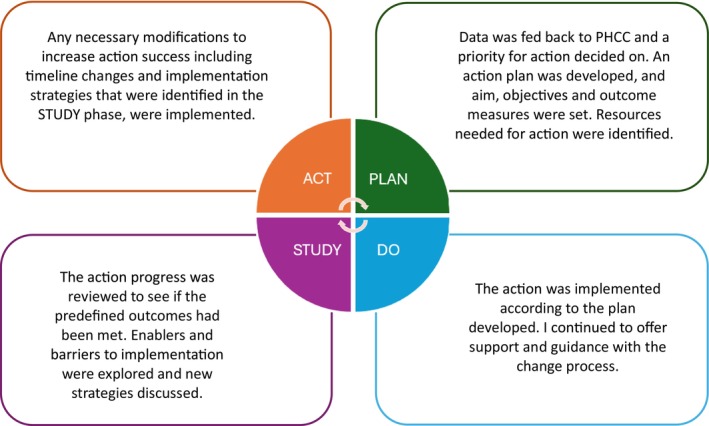
Plan–Do–Study–Act cycle.

Findings from the yarning circles and audit results informed selection of continuous quality improvement (CQI) projects for Cycle 3. Plan–Do–Study–Act (PDSA) cycles, a four‐stage problem‐solving method [16], were implemented to address identified priorities. The implementation process at each PHCC is summarised in Figure 2.

Plan–Do–Study–Act cycles identified by clinic staff were implemented iteratively from November 2022 to April 2025, with some continuing beyond the project period. Timelines and priority actions varied between PHCCs. Consistent with a decolonising approach, staff determined and adjusted these actions in response to contextual factors within the PHCC and broader community.

Cycle 4 identified principles and action strategies arising from yarning circle findings, audit data and CQI activities. These were incorporated into a draft Ageing Well Framework. A final workshop was held at each of the five PHCCs to discuss findings and refine the Framework. This draft framework was disseminated to relevant stakeholders across the region, including the Knowledge Circle, and all input and feedback were synthesised into the final framework.

## 3 Results

### 3.1 Clinical Audits

#### 3.1.1 Patient Demographics

Patient demographics are presented in Table 1. The mean age was 42.24 years (SD = 16.3, range 18–95 years), with 26% of the sample (*n* = 288) over 55 years of age. Within the 19% of the sample (*n* = 210) who did not identify as Aboriginal and/or Torres Strait Islander, ethnicities recorded included Papua New Guinean, Māori and Pacific Islander. As these population groups potentially share similar comorbidities and problems associated with ageing, these data were aggregated into the overall data. Data from a small number of Caucasians also living in the region were included, as the overall framework was designed for all residents in the Torres Strait.

**TABLE 1:** Characteristics of audit participants.

	Site 1 (*n* = 158), *n* (%)	Site 2 (*n* = 391), *n* (%)	Site 3 (*n* = 160), *n* (%)	Site 4 (*n* = 119), *n* (%)	Site 5 (*n* = 300), *n* (%)	Total (*n* = 1128), *n* (%)
**Age**						
Mean (SD)	41.28 (17.83)	40.29 (15.74)	48.19 (17.02)	42.01 (14.67)	42.22 (15.75)	42.24 (16.3)
Range	18–95	18–82	19–83	19–92	19–81	18–95
< 55 years	124 (79)	303 (78)	96 (60)	96 (81)	221 (74)	840 (75)
≥ 55 years	34 (22)	88 (23)	64 (40)	23 (19)	79 (26)	288 (26)
**Sex**						
Male	76 (48)	200 (51)	74 (46)	59 (50)	128 (43)	537 (48)
Female	82 (52)	191 (49)	86 (54)	60 (50)	172 (57)	591 (52)
**Indigenous status**						
Torres Strait Islander	136 (86)	182 (47)	137 (86)	117 (98)	210 (70)	782 (69)
Aboriginal	0 (0)	10 (3)	6 (4)	0 (0)	5 (2)	21 (2)
Both^a^	15 (10)	43 (11)	7 (4)	1 (1)	44 (15)	110 (10)
Neither	7 (4)	153 (39)	10 (6)	1 (1)	39 (13)	210 (19)
Not specified^b^	0 (0)	3 (1)	0 (0)	0 (0)	2 (1)	5 (0)
**Marital status**						
Single	15 (10)	45 (12)	25 (16)	16 (13)	54 (18)	155 (14)
Married	20 (13)	39 (10)	37 (23)	18 (15)	41 (14)	155 (14)
De facto	26 (17)	48 (12)	15 (9)	13 (11)	51 (17)	153 (14)
Widowed	6 (4)	2 (1)	5 (3)	4 (3)	11 (4)	28 (3)
Divorced	4 (3)	3 (1)	2 (1)	0 (0)	4 (1)	13 (1)
Not specified^b^	87 (55)	254 (65)	76 (48)	68 (57)	139 (46)	624 (55)

^a^ Both Aboriginal and Torres Strait Islander.

^b^ No record of Indigenous status in the patient chart.

Audit data from across the 12 domains are presented as Supporting Information tables (see Tables S1–S17). Key audit results included:

#### 3.1.2 Chronic Disease

High rates of chronic disease, particularly in the over 55 years age group, were documented. As shown in Table 2, almost 70% of the over 55s had diabetes, 22% had coronary artery disease, and 60% had hypertension.

**TABLE 2:** Documented chronic disease diagnoses.

Diagnosis	< 55 years (*n* = 840), *n* (%)	≥ 55 years (*n* = 288), *n* (%)	Total (*n* = 1128), *n* (%)
Diabetes	292 (35)	198 (69)	490 (43)
Coronary artery disease^a^	27 (3)	62 (22)	89 (8)
Dyslipidaemia	127 (15)	157 (55)	284 (25)
Stroke/TIA	8 (1)	20 (7)	28 (3)
Chronic kidney disease	62 (7)	119 (41)	181 (16)
Hypertension	98 (12)	173 (60)	271 (24)
Atrial fibrillation	8 (1)	16 (6)	24 (2)
Rheumatic heart disease	47 (6)	10 (4)	57 (5)
Congestive heart failure	2 (0.2)	9 (3)	11 (1)

Abbreviation: TIA, transient ischaemic attack.

^a^ Included documentation of either cardiovascular disease/ischemic heart disease/coronary artery disease/coronary heart disease.

Over 53% had three or more chronic diseases, and 30 people (10%) had five or more of the chronic diseases listed. Only 14% had no chronic diseases documented. Despite the high prevalence of chronic disease, only 80 clients overall (7%) and 33 individuals aged 55 years or older (12%) had evidence of a cardiovascular risk (CVR) assessment within this period. However, of those assessed as having a CVR of > 10%, there was a HSR in all cases of those aged younger than 55 years and in 56% of those aged 55 years or older.

#### 3.1.3 Dementia Risk Factors

The HAAT data included the following 11 risk factors from the 2024 Lancet Commission potentially modifiable risk factors for dementia: [17] hearing loss, hypertension, smoking, depression, physical inactivity, obesity, diabetes, traumatic brain injury, excessive alcohol consumption, vision loss and high cholesterol. Data were not available to assess the other three factors—social isolation, air pollution and education levels. Over 77% had at least one of the Lancet risk factors for dementia, with 14% having four or more risk factors. Some risk profiles may have been higher due to missing data on smoking, alcohol and physical activity.

#### 3.1.4 Clinical Measurements

Key clinical indicators of cardiovascular disease, diabetes and chronic kidney disease risk are shown in Table 3. The HSR response to abnormal readings, as per the National Guide to a Preventive Health Assessment for Aboriginal and Torres Strait Islander Peoples [13] was also audited.

**TABLE 3:** BP, ACR, HbA1C, eGFR and lipid profile readings and HSR.

Domain	< 55 years (*n* = 840), *n* (%)	≥ 55 years (*n* = 288), *n* (%)	Total (*n* = 1128), *n* (%)
**BP reading**	680 (81)	259 (89.9)	939 (83.2)
Systolic reading of ≥ 140	119 (17.5)	91 (35.1)	
HSR to systolic reading of ≥ 140	36 (30)	36 (39.5)	
**ACR reading**	299 (35.6)	142 (49.3)	441 (39.1)
Raised ACR^a^	98 (32.7)	74 (52.1)	
HSR to raised ACR	60 (61.2)	50 (67.5)	
**HbA1C**	432 (51.4)	195 (67.7)	627 (55.6)
Abnormal profile^b^	90 (20.8)	90 (46.2)	
HSR to abnormal profile	71 (78.8)	78 (86.7)	
**eGFR reading**	488 (58.09)	231 (80.2)	719 (63.7)
Low eGFR^c^	20 (4.1)	61 (26.4)	
HSR to low eGFR	12 (60)	40 (65.6)	
**Lipid profile test**	413 (49.2)	200 (69.4)	613 (54.3)
Abnormal profile^d^	358 (86.7)	173 (86.5)	
HSR to abnormal profile	122 (34.1)	61 (35.3)	

^a^ Raised ACR is > 2.5 mg/mmol (male) or > 3.5 mg/mmol (female).

^b^ HbA1C abnormal > 53 mmol/7%.

^c^ Low eGFR is < 60 mL/min/1.73 m^2^.

^d^ LDL‐C > 2.5 mmol/L OR HDL‐C < 1.0 mmol/L OR Triglycerides > 1.5 mmol/L OR Total cholesterol/HDL ratio > 4.5 mmol/L.

Most markers for chronic disease were being monitored, notably blood pressure, with the majority of the sample having a measurement within the previous 12 months. The number of patients with abnormal results was higher in the over 55 year's old group, reflecting the impact of midlife chronic disease, with the greatest HSR focused on addressing diabetes and kidney function.

#### 3.1.5 Physical Activity, Nutrition and Obesity

The HAAT audit documented concerns about physical activity, obesity and nutrition (including malnutrition and food access), and recorded weight, height and body mass index (BMI; Supporting Information Tables). Physical activity (PA) assessment was low, with only 28% (*n* = 314) having PA documented. Although the 715HA includes a PA question, no validated screening tool was used. When PA concerns were noted, an HSR occurred in 37% (*n* = 43) of cases.

The 715HA includes questions on nutrition and food access but no validated nutrition or obesity screening tools. The HAAT captured obesity concerns from clinical notes; weight, height and BMI were audited but not assumed to be used for screening. Obesity concerns were documented in 17% (*n* = 194) of the sample, and when concerns were raised an HSR occurred in 96% (*n* = 187).

#### 3.1.6 Vision and Hearing

Eye screening (visual acuity [VA], fundal examination, relative afferent pupillary defect, intraocular pressure, fundoscopy and extraocular muscle testing) to identify retinopathy, glaucoma, age‐related macular degeneration, cataracts and trichiasis were audited. Overall, 33% (*n* = 377) received at least one test, with VA—a routine component of the 715HA—most frequently performed (*n* = 371). All but one identified concern resulted in an HSR.

Hearing screens were documented for 5% (*n* = 53) of clients. Of those screened, 64% (*n* = 34) had concerns raised, and all received an appropriate HSR.

#### 3.1.7 Mood Disorders

The HAAT recorded diagnoses of depression and anxiety, evidence of Social and Emotional Wellbeing (SEWB) screening, tools used and any HSRs. A range of mental health tools was used, including adaptations of existing measures and general SEWB questions within the 715HA. Overall, 30% (*n* = 343) received a mood or SEWB screen through general questioning or a formal tool. Among those screened, 30% (*n* = 102) had identified concerns, and 93% (*n* = 95) resulted in an HSR.

#### 3.1.8 Cognitive Functioning

Of the 474 patients aged 45 and over, 19% (*n* = 90) were screened for cognition. Concerns were raised about cognition with 7% (*n* = 35) of patients. Of the 59 patients assessed using a standardised tool, 27 were assessed using the KICA‐Cog [18] (the only validated tool for Aboriginal and Torres Strait Islander Peoples).

#### 3.1.9 Functional Status

Functional assessments were conducted with 21% (*n* = 60) of clients aged 55 years or older. Of these, 60% (*n* = 36) occurred within the 715HA conducted by GPs (*n* = 34) or Indigenous Health Workers (IHW; *n* = 2). Outside the 715HA, assessments were completed by Allied Health professionals (*n* = 11) or visiting geriatric services (*n* = 13). Functional concerns were identified for 10 clients (4% of over 55 s) of whom 90% (*n* = 9) received an HSR.

#### 3.1.10 715HA

At the time of the initial audit, completed 715HA were found in 18% (*n* = 151) of those younger than 55 years and 26% (*n* = 76) of those 55 years or older.

### 3.2 CQI Activities

#### 3.2.1 Site Specific Workshops

Between three and seven workshops were held at each of the participating PHCCs. Details of the frequency of workshops and staff attending can be found in Supporting Information. Staff included: IHWs, clinical nurses (Indigenous and non‐Indigenous), practice managers, program directors, outreach team leaders, General Practitioner (GP), PHCC administration officers and Allied Health clinicians.

#### 3.2.2 CQI Activities

Some CQI activities were unique to individual PHCCs, but many were shared across sites, with all five sites using their own data to identify and prioritise issues. However, because all PHCCs operated under the one health service governance, many initiatives had broader impacts, leading to changes implemented across multiple sites. Key activities are outlined in Table 4.

**TABLE 4:** CQI activities undertaken.

Developing a consistent template for the 715HA, and increasing completion rates. Increasing billing for Medicare items. Increasing patient involvement in the 715HA with inclusion of IHW in the GP consult. Lay communication with the patient after consults to explain outcomes and plans. Increasing screening for falls, continence, mood and cognition in those ≥ 55 years. A more holistic focus to the 715HA with increased screening for social and cultural determinants of health (SCDoH), such as SEWB, social engagement and cultural connections. Use of validated culturally appropriate screening tools specifically for cognition, SEWB, diet and PA. Exploring use of ‘red flags’ to identify decline and frailty. Environmental changes to facilitate sensitive yarning topics with clients. Exploring collaboration with other community groups, Community Controlled Health Organisations, Councils, Universities Non‐Government Organisations and aged care providers to increase access to educational/information events, and social and activity programs. Increasing focus on health promotion and chronic disease prevention. Increasing health and community awareness of dementia risk factors. Increasing access and use of Allied Health services, and an increase in functional assessments. Sourcing of equipment, such as portable centrifuge and defibrillator. Increasing hearing screens, and foot checks. Increasing use of IHWs and Nurse Practitioners working to full scope of practice.

### 3.3 The Ageing Well Framework

The final co‐designed Framework can be accessed at https://doi.org/10.25903/0se8‐vb72. The Framework sets out recommendations and strategies at three levels: Community, Primary Health Care and Individual.

#### 3.3.1 Community Level

The Framework outlines how age‐friendly environments that incorporate accessible housing, subsidised transport and safe, well‐maintained public spaces can support independence, mobility and physical activity. Economic and workforce components that enable financial security and sustainable service delivery are also included. Social connectedness is emphasised, including physical activity opportunities, nutrition and cooking initiatives, social groups and culturally grounded activities that promote physical, mental and social well‐being. Cultural engagement is essential. The Framework includes activities that support connection to language, traditions and respect for Elders, alongside intergenerational initiatives that foster cultural continuity, mutual learning and shared purpose. Service delivery components within the Framework outline the need for flexible aged care models, expanded respite options and enhanced support for informal carers through training, respite and peer networks. The Framework also includes opportunities for community education and accessible service information to improve navigation of available supports.

#### 3.3.2 Primary Health Care Level

The Framework prioritises health promotion and chronic disease prevention through life course approaches, incorporation of preventive advice in routine care, culturally appropriate screening and increased use of comprehensive 715HAs. There is an emphasis on patient and family‐centred care, including active involvement in decision‐making, adequate time for holistic assessments and the centralising of IHWs to enhance understanding and cultural safety. Holistic, strengths‐based assessments addressing physical, cognitive, functional, cultural and social domains are key. The Framework outlines strategies to ensure equitable, culturally competent and evidence‐based care, including expanded specialist access, integrated digital systems and use of validated screening tools. Dementia, frailty and functional decline are important priorities, with early detection and targeted interventions recommended. Advance care planning, culturally responsive palliative care, full utilisation of the existing workforce and strengthened research partnerships are also integral components.

#### 3.3.3 Individual Level

The Framework emphasises the importance of connection to family, friends and community, as well as cultural connections, as a foundation for ageing well. The Framework highlights traditional ways of living as protective, including regular physical activity, healthy diets that include local foods and lifelong learning. Social and emotional well‐being is supported through meaningful roles, spiritual practices, self‐care and access to mental health services, with resilience and adaptability recognised as important capacities. Autonomy and health literacy are presented as essential for maintaining independence, enabling informed decision‐making and effective self‐management. Strategies to build health literacy and self‐management skills include clear communication, involvement of IHWs and family, technology use and regular health monitoring.

The Framework also includes a toolbox of resources, including culturally validated tools to screen for cognition, SEWB, quality of life, diet and PA, locally specific information to support carers, and links to national resources to support the health, well‐being and ageing of Aboriginal and Torres Strait Islander Peoples.

## 4 Discussion

Preventable chronic diseases continue to contribute to health inequities for Aboriginal and Torres Strait Islander Peoples, with Cardio Vascular Disease (CVD) a major contributor to premature mortality [19, 20]. Audit findings reflected this burden, showing high multimorbidity among adults 55 years or older and substantial CVD prevalence, yet documentation of CVR assessments was minimal. This mirrors national evidence where CVR screening remains low, largely due to poor recording of individual risk factors, such as smoking, alcohol use and physical activity [20]. Embedding automated CVR calculators within electronic medical records and integrating CVR assessment into 715HAs have been proposed to increase screening rates [20].

Audits also revealed limited follow‐up for elevated CVR and underuse of chronic disease management plans, consistent with barriers identified elsewhere, including acute care workload, workforce limitations and inconsistent adherence to guidelines [20]. Strengthening the role of IHWs in chronic disease management is warranted to address broader social and cultural determinants of health influencing lifestyle behaviours [21]. Improving chronic disease care requires Indigenous community co‐design, a trained and adequately supported workforce, strong therapeutic relationships, clear clinical pathways and culturally safe, coordinated models of care [22]. These elements are essential for enhancing CVD prevention and chronic disease management.

The 715HAs were established to support early detection and prevention of chronic disease [19], yet this study identified low screening rates for chronic disease risk factors and ageing‐related conditions. Primary Health Care staff, through the CQI activities, acknowledged that issues associated with the content of the 715HA were comparable to those reported elsewhere, including a lack of inclusion of the social and cultural determinants of health and patient‐identified issues, along with perceptions of their superficiality [19]. Consistent with national data [23], 715HA uptake was low, representing missed opportunities to strengthen preventive care, enhance IHW involvement and embed culturally appropriate screening and management planning.

Audit findings demonstrated low levels of Allied Health (AH) involvement across participating primary healthcare centres, despite AH clinicians being recognised as integral to effective chronic disease management [24]. Allied Health supports prevention, early intervention and rehabilitation, with early identification of risk factors essential for reducing long‐term disease burden. Increasing AH rebate utilisation could help offset staffing costs and enhance service availability in remote regions. A newly implemented AH student‐led program delivered in one Torres Strait community offers a cost‐effective approach to expanding AH services. Although not solely focused on chronic disease, the program promotes PA, mobility, social connection and culturally responsive care, aligning with preventative health priorities. The model has demonstrated feasibility and cultural relevance in Cape York communities [25] and could be scaled across the Torres Strait and NPA.

Lifestyle behaviours strongly influence chronic disease and dementia risk, shaping overall ageing outcomes. Audit results indicated limited screening for PA and nutrition consistent with national trends of reports of low PA and suboptimal dietary patterns among Indigenous adults [26]. This is despite PA and obesity being major modifiable risk factors [27], highlighting a missed opportunity for early intervention. The absence of validated tools for assessing diet and weight management further constrains preventative care, particularly in regions where food insecurity significantly shapes nutritional behaviour. Effective prevention requires culturally appropriate, strengths‐based screening approaches and integration of Indigenous knowledges, including traditional foods and community‐led nutrition programs. Strengthening screening systems, embedding culturally validated tools and supporting community‐led initiatives are critical steps towards addressing the underlying determinants of chronic disease in the region.

Vision and hearing impairments are established risk factors for dementia [17]. Either condition can reduce functional capacity and quality of life, particularly by limiting social engagement and participation in community activities. Audit results showed only 4% of participants received a formal hearing assessment in the previous year and 33% underwent a vision check. These findings align with earlier evidence that routine eye and vision assessments for Aboriginal and Torres Strait Islander adults in primary health care settings remain well below recommended levels [28]. Sensory health concerns have also been highlighted in longitudinal research on healthy ageing in an Indigenous cohort in the Kimberley, where declines in vision and hearing were identified as significant issues [23]. Consistent with these findings, the authors have advocated for enhanced incorporation of vision and hearing screening within the 715HA to support healthier ageing outcomes.

Dementia diagnosis requires evidence of cognitive impairment, functional decline, informant corroboration, documented change in cognition and exclusion of reversible causes [29]. Best practice recommends detection through symptoms/concerns raised, risk‐factor assessment and the use of cognitive screening tools [29]. However, HAAT data indicated limited implementation. Only 19% of clients aged 45 years or older (*n* = 474) had any cognitive screening in the previous 12 months, with most formal screenings conducted by visiting geriatric specialists rather than PHC staff. This low screening rate is concerning given the high regional prevalence of cognitive impairment in the Torres Strait and NPA [4], and the recommendation for active case finding and routine cognitive screening from age 50 years in Aboriginal and Torres Strait Islander populations [29]. Tool selection was a further issue, with the KICA‐cog used infrequently by the PHCC clinicians.

Mental health concerns among Indigenous populations, particularly older adults, remain a significant issue [30], with depression a recognised risk factor for dementia [17]. Both the yarning circles and CQI activities emphasised SEWB as a central determinant of healthy ageing. The HAAT findings are concerning, as fewer than 20% of clients received any documented mood screening via structured tools or informal questioning. Screening practices were inconsistent, with multiple tools and multiple versions of the same tool used during the 715HA, creating variable interpretation. Primary Health Care staff also noted that these tools did not adequately capture culturally relevant SEWB domains or social isolation, another dementia risk factor. Assessing SEWB in Indigenous populations remains challenging due to the limited availability of culturally appropriate tools [31]. Standard depression and anxiety measures, grounded in Western biomedical models, fail to capture Indigenous conceptualisations of well‐being and the central role of culture [32]. Although a SEWB screening tool has been developed for Aboriginal communities in Western Australia [31], an equivalent remains in the process of validation by HART for Torres Strait Islander Peoples. Additionally, conducting SEWB assessments in busy clinical environments with limited time was reported as a barrier to sensitive and culturally meaningful screening. Creating safe yarning spaces enhances culturally safe care, an essential factor given culturally unsafe PHC environments are a documented barrier to care for Aboriginal and Torres Strait Islander Peoples [19].

The use of CQI is well established as an effective approach for strengthening preventative screening, 715HA delivery, follow‐up of abnormal results and evidence‐based care in Indigenous PHC [19]. Although several CQI activities were identified as priorities, PHCC staff in this study encountered barriers to implementing CQI, such as workforce instability, acute workloads and limited medical engagement, consistent with challenges reported elsewhere, whereas facilitators included stable staffing, strong leadership and coherent teams [33]. Staff also highlighted unmet needs beyond health services, such as lack of transport, social programs and carer support, highlighting the necessity of multisector collaboration to support ageing well.

## 5 Limitations

The clinical audit findings were limited by reliance on documented data in electronic records, which may underestimate actual care—a limitation also noted in previous CQI studies [19]. Only clients with clinical contact in the previous 12 months were included, potentially excluding less‐engaged individuals or those who received additional care through the local Community Controlled Health Service and thereby potentially biasing results. Aggregated data included a number of residents in the region who did not identify as Aboriginal and/or Torres Strait Islander. Although many of these were of other Indigenous backgrounds and therefore likely sharing similar comorbidities and problems, others were not. This limits interpretation of the data when applying results to Aboriginal and Torres Strait Islander populations. However, all data were included as the framework was designed to support all adults living in the region. Additionally, the HAAT, although aligned with best practice guidelines, was not tested for reliability or validity, further constraining interpretation of audit outcomes.

## 6 Conclusions

Ageing well in the Torres Strait and NPA is shaped by a holistic view of health and impacted by chronic disease, functional and cognitive health, socioemotional well‐being and broader social and cultural determinants of health. Continuous quality improvement activities highlighted gaps in screening, follow‐up and culturally appropriate assessment within PHC services, alongside clear opportunities to strengthen interdisciplinary care, expand IHW roles and embed culturally grounded screening tools. At the same time, many priorities identified extended beyond the scope of health services alone, reinforcing the need for coordinated, multisector strategies.

The principles and action strategies identified through this work informed the development of the Framework that emphasised a whole‐of‐community response. Bringing together health services, councils, non‐government organisations, aged care providers, academia and the private sector, the Framework offers evidence‐based guidance for community, PHC and individual levels. These include building age‐friendly environments, improving integration across health, social and aged care sectors, strengthening community‐based programs, and supporting intergenerational connections. At the PHC level, system improvements are proposed to enable culturally appropriate, best practice gerontic care. At the individual level, the Framework foregrounds cultural determinants of health as protective factors that support people to maintain cultural, physical, cognitive and social functioning. This Ageing Well Framework can be used to support older Aboriginal and Torres Strait Islander Peoples in the region to flourish, remain connected and continue living at home and in their communities, an aspiration shared by many and central to ageing well in place.

## Ethics Statement

Ethics approval was obtained from the Far North Queensland Human Research Ethics Committee (HREC/2020/QCH/59342–1406) and the James Cook University Human Research Ethics Committee (H8063).

## Conflicts of Interest

The authors declare no conflicts of interest.

## Supporting information


**Table S1:** 715 Aboriginal and Torres Strait Islander Health Assessments.
**Table S2:** Documented Cardiovascular Risk Assessment and HSR.
**Table S3:** Lancet Modifiable Risk Factors for Dementia.
**Table S4:** Smoking and Alcohol Consumption Status and HSR.
**Table S5:** Polypharmacy and Medication Reviews.
**Table S6:** Concerns Raised about Physical Activity and Obesity and HSR.
**Table S7:** Billing of services.
**Table S8:** Vision and Hearing Screening and HSR.
**Table S9:** Osteoporosis Screening and HSR.
**Table S10:** Foot and dental checks.
**Table S11:** Continence screening and HSR.
**Table S12:** Mood disorders, SEWB Screening and HSR.
**Table S13:** Cognitive screening and HSR for clients aged 45 years or older (*n* = 474).
**Table S14:** EPOA, ACP and EOL care discussions or completed paperwork for clients older than 55 years (*n* = 288).
**Table S15:** Functional assessment, concerns raised and Allied Health input for clients older than 55 years (*n* = 288).
**Table S16:** Support services and social engagement.
**Table S17:** Details of the number and composition of the face‐to‐face workshops.

## Data Availability

The data that support the findings of this study are available on request from the corresponding author. The data are not publicly available due to privacy or ethical restrictions.
