# Supplementary material for: The Development of a Framework to Support Ageing Well in the Torres Strait and Northern Peninsula Area of Australia
Source: Australas J Ageing. 2026 Jul 27;45(3):e70211. doi: 10.1111/ajag.70211 (PMC13404143; doi:10.1111/ajag.70211)
Supplement: Supplementary file 1 — Table S1: 715 Aboriginal and Torres Strait Islander Health Assessments. Table S2: Documented Cardiovascular Risk Assessment and HSR. Table S3: Lancet Modifiable Risk Factors for Dementia. Table S4: Smoking and Alcohol Consumption Status and HSR. Table S5: Polypharmacy and Medication Reviews. Table S6: Concerns Raised about Physical Activity and Obesity and HSR. Table S7: Billing of services. Table S8: Vision and Hearing Screening and HSR. Table S9: Osteoporosis Screening and HSR. Table S10: Foot and dental checks. Table S11: Continence screening and HSR. Table S12: Mood disorders, SEWB Screening and HSR. Table S13: Cognitive screening and HSR for clients aged 45 years or older (n = 474). Table S14: EPOA, ACP and EOL care discussions or completed paperwork for clients older than 55 years (n = 288). Table S15: Functional assessment, concerns raised and Allied Health input for clients older than 55 years (n = 288). Table S16: Support services and social engagement. Table S17: Details of the number and composition of the face‐to‐face workshops. [file AJAG-45-0-s001.docx]

**Supplementary tables**

**Supplementary Table 1:** 715 Aboriginal and Torres Strait Islander Health Assessments

|  | **Site 1 (n = 158)**  **n (%)** | **Site 2 (n = 391)**  **n (%)** | **Site 3 (n = 160)**  **n (%)** | **Site 4 (n = 119)**  **n (%)** | **Site 5 (n = 300)**  **n (%)** | **Total (n = 1,128)**  **n (%)** |
| --- | --- | --- | --- | --- | --- | --- |
| **715HA completed** |  |  |  |  |  |  |
| <55 years | 21 (17) | 18 (6) | 15 (16) | 49 (51) | 48 (22) | 151 (18) |
| >55 years | 5 (15) | 7 (8) | 19 (30) | 15 (65) | 30 (38) | 76 (26) |
| **Partial 715HA completed** |  |  |  |  |  |  |
| <55 years | 5 (4) | 17 (6) | 9 (9) | 21 (22) | 21 (10) | 73 (9) |
| >55 years | 1 (3) | 16 (18) | 7 (11) | 5 (22) | 10 (13) | 39 (14) |

**Supplementary Table 2:** Documented Cardiovascular Risk Assessment and HSR

|  | **<55 years (n = 840)**  **n (%)** | **>55 years (n = 288)**  **n (%)** | **Total (n = 1,128)**  **n (%)** |
| --- | --- | --- | --- |
| **Record of CVR assessment** | 47 (6) | 33 (12) | 80 (7) |
| Score of >10 | 3 (16) | 16 (84) | 19 (2) |
| Documented HSR | 3 (100) | 9 (56) | 12 (63) |

HSR – Health Service Response

**Supplementary Table 3:** Lancet Modifiable Risk Factors for Dementia

| **No of risk factors** | **<55 years (n = 840)**  **n (%)** | **>55 years (n = 288)**  **n (%)** | **Total (n = 1,128)**  **n (%)** |
| --- | --- | --- | --- |
| 0 | 231 (28) | 22 (8) | 253 (22) |
| 1 | 241 (29) | 43 (43) | 284 (25) |
| 2 | 192 (23) | 63 (22) | 255 (23) |
| 3 | 108 (13) | 75 (26) | 183 (16) |
| 4 | 53 (6) | 60 (21) | 113 (10) |
| 5 | 12 (1) | 19 (7) | 31 (3) |
| 6 | 3 (0) | 3 (1) | 6 (1) |
| 7 | 0 (0) | 3 (1) | 3 (0) |

**Supplementary Table 4:** Smoking and Alcohol Consumption Status and HSR

| **Domain/HSR** | **n (%)** | **Not recorded** |
| --- | --- | --- |
| Current smoker | 414 (37) | 129 (11) |
| HSR | 194 (47) |  |
| High alcohol consumption | 95 (8) | 328 (29) |
| HSR | 52 (55) |  |

HSR – Health Service Response

**Supplementary Table 5:** Polypharmacy and Medication Reviews

| **Domain** | **<55 years (n=840)**  **n (%)** | **>55 years (n=288)**  **n (%)** | **Total (n= 1,128)**  **n (%)** |
| --- | --- | --- | --- |
| Polypharmacy | 76 (9) | 156 (54) | 232 (21) |
| Medication review | 190 (23) | 158 (55) | 348 (31) |
| N/A for medication review | 239 (29) | 14 (5) | 253 (22) |

**Supplementary Table 6:** Concerns Raised about Physical Activity and Obesity and HSR

| **Domain** | **n (%)** |
| --- | --- |
| **Physical activity** | 314 (28) |
| Concerns raised | 115 (37) |
| HSR to concerns raised | 43 (37) |
| **Obesity** | 0 (0) |
| Concerns raised | 194 (17) |
| HSR to concerns raised | 187 (96) |

HSR – Health Service Response

**Supplementary Table 7:** Billing of services

| **Item numbers** | **Service** | **n (%)** |
| --- | --- | --- |
| 715 | Adult Health Check | 284 (25) |
| 721 | GP management plan (GPMP) | 171 (15) |
| 723 | Team care arrangements (TCA) | 152 (14) |
| 732 | Review of GPMP or TCA | 56 (5) |
| 900 | Domiciliary Medication Management Review | 0 (0) |
| 2700-2717 | Mental health planning | 12 (1) |
| 10987 | Follow-up from nurse or IHW | 12 (1) |
| 82200-82215 | Nurse Practitioner | 0 (0) |
| 81300 | IHW | 0 (0) |
| 10951-10970/  81315-81360 | Allied health | 54 (5) |
| 81305 | Diabetes Educator | 2 (0) |
| 81310 | Audiologist | 0 (0) |
|  | No claims | 745 (66) |

IHW – Indigenous Health Workers

**Supplementary Table 8:** Vision and Hearing Screening and HSR

| **Domain** | **n (%)** |
| --- | --- |
| **Vision** | 377 (33) |
| Concerns raised | 84 (22) |
| HSR to concerns raised | 83 (99) |
| **Hearing** | 53 (5) |
| Concerns raised | 34 (64) |
| HSR to concerns raised | 34 (100) |

HSR – Health Service Response

**Supplementary Table 9: Osteoporosis Screening and HSR**

| **Domain** | **n (%)** |  | **N/A*** | **Unknown menopausal status^**^** |
| --- | --- | --- | --- | --- |
| **Osteoporosis screening** | 5 (0) |  | 739 (66) | 93 (8) |
| Client deemed high risk | 2 (40) |  |  |  |
| HSR to high-risk clients | 2 (100) |  |  |  |

* N/A for clients not in correct age range—osteoporosis screening is recommended for all postmenopausal women and men over the age of 50 years as per the *National Guide to preventive health assessment for Aboriginal and Torres Strait Islander People* (National Aboriginal Community Controlled Health Organisation and The Royal Australian College of General Practitioners, 2018).

** Unknown from medical record if client is postmenopausal.

HSR – Health Service Response

**Supplementary Table 10:** Foot and dental checks

| **Domain** | **<55 years (n = 840)**  **n (%)** | **>55 years (n = 288)**  **n (%)** | **Total (n = 1,128)**  **n (%)** |
| --- | --- | --- | --- |
| Foot check | 65 (8) | 61 (21) | 126 (11) |
| Dental check | 65 (8) | 26 (9) | 91 (8) |

**Supplementary Table 11:** Continence screening and HSR

| **Domain** | **<55 years (n = 840)**  **n (%)** | **>55 years (n = 288)**  **n (%)** | **Total (n = 1,128)**  **n (%)** |
| --- | --- | --- | --- |
| **Continence screen** | 101 (12) | 76 (26) | 177 (16) |
| Concerns raised | 6 (6) | 20 (26) | 26 (15) |
| HSR to concerns raised | 4 (67) | 15 (75) | 19 (73) |

HSR – Health Service Response

**Supplementary Table 12:** Mood disorders, SEWB Screening and HSR

| **Domain** | **n (%)** |
| --- | --- |
| **Depression diagnosis** | 69 (6) |
| **Anxiety diagnosis** | 2 (0) |
| **Mood/SEWB screening**  Standard tool  Questions only  Both | 183 (16)  91 (8)  69 (6) |
| Concerns raised | 102 (30) |
| HSR to concerns raised | 95 (93) |

HSR – Health Service Response

SEWB -- Social and Emotional Wellbeing

**Supplementary Table 13:** Cognitive screening and HSR for clients aged 45 years or older (n = 474)

| **Domain** | n **(%)** |
| --- | --- |
| **Cognition screen** | 90 (19) |
| **Screening by**  General questions by GP in consult  Questions in the AHC  Screening tool | 5 (6)  33 (37)  59 (66) |
| Concerns raised | 35 (7) |
| HSR to concerns raised |  |
| Referred to a specialist | 43 (9) |
| Further investigations^*^ carried out | 6 (17) |

HSR – Health Service Response

**Supplementary Table 14:** EPOA, ACP and EOL care discussions or completed paperwork for clients older than 55 years (n = 288)

| **Domain** | **n (%)** |
| --- | --- |
| EPOA appointed or discussed | 15 (5) |
| EOL care discussed | 0 (0) |
| ACP /ACD discussed or in place | 19 (7) |

EPOA – Enduring Power of Attorney

EOL – end-of-life

ACP – Advanced Care Planning

ACD – Advanced Care Directive

**Supplementary Table 15:** Functional assessment, concerns raised and Allied Health input for clients older than 55 years (n = 288)

| **Domain** | **n (%)** |
| --- | --- |
| **Evidence of a functional assessment** | 60 (21) |
| Assessment as part of the AHC | 36 (60) |
| **Components of functional assessment documented** |  |
| Personal ADLs | 60 (21) |
| Instrumental ADLs | 60 (21) |
| Pain | 19 (7) |
| Falls | 35 (12) |
| Financial capacity | 13 (5) |
| Driving | 24 (8) |
| Mobility | 6 (2) |
| Continence | 3 (1) |
| Concerns raised | 10 (17) |
| HSR to concerns raised | 9 (90) |
| **Occasions of service of AH** |  |
| Podiatrist | 58 (20) |
| Physiotherapist | 43 (15) |
| Dietitian | 42 (15) |
| Diabetes Educator^*^ | 29 (10) |
| Occupational Therapist | 24 (8) |
| Pharmacist | 7 (2) |
| Speech Pathologist | 3 (1) |
| Social Worker | 3 (1) |
| Psychologist | 0 (0) |
| **Type of service delivered** |  |
| Weight management/nutritional support | 19 (7) |
| Pain management | 29 (10) |
| Activity program | 0 (0) |
| Medication management | 7 (2) |
| Housing, Aged Care, NDIS referrals | 2 (1) |
| Home visit assessment, modifications and equipment | 20 (7) |
| Provision of mobility aids | 5 (2) |
| Foot care | 58 (20) |
| Diabetes education and management | 40 (14) |
| Falls prevention | 3 (1) |
| Pelvic floor exercises | 1 (0) |
| Speech and swallowing therapy | 3 (1) |
| Hand therapy | 1 (0) |
| Stroke rehabilitation | 1 (0) |
| Pulmonary rehabilitation | 1 (0) |
| Compression therapy | 1 (0) |
| Vestibular therapy | 1 (0) |
| **Filled by role of nurse within the health service |  |

**Supplementary Table 16:** Support services and social engagement

| **Domain** | **n (%)** |
| --- | --- |
| RAS or ACAT assessment | 18 (6) |
| Evidence of receiving Aged Care services | 11 (4) |
| Evidence of receiving NDIS support | 5 (1) |
| Evidence of social engagement through: |  |
| Church | 4 (0) |
| Family | 12 (1) |
| Community groups | 1 (0) |
| Cultural events | 1 (0) |
| CHSP/HCP—group activity | 1 (0) |
| Social organisation/club | 1 (0) |
| Sporting organisation/club | 6 (1) |
| School club | 1 (0) |
| Army Reserves | 1 (0) |

**Supplementary Table 17:** Details of the number and composition of the face-to-face workshops

| **Site** | **Number of workshops** | **Staff involved** |
| --- | --- | --- |
| 1* | Seven | - TCHHS Primary Health Care Program Director - Indigenous Practice Manager (PM) of the PHCC that housed the outreach team on a different island - Staff of the outreach team   - IHW outreach team leader   - Indigenous Clinical Nurse (CN)   - 2 IHWs   - 2 non-Indigenous CNs |
| 2 | Five | - Indigenous PM - 3 IHWs - 2 non-Indigenous CNs—one of whom has lived and   worked on the island for several decades   - regular visiting non-Indigenous GP - Indigenous admin officer of the PHCC |
| 3 | Five | - Indigenous PM - 1 IHW - 1 non-Indigenous NP who has worked at the PHCC and lived on the island with her family for several decades |
| 4** | Three | - 2 IHWs - 1 non-Indigenous locum CN |
| 5 | Six | - TCHHS Indigenous Assistant Director of Nursing Workforce Design - TCHHS Primary Health Care Program Director - Indigenous Island Cluster Manager for the region (whose position manages the PM) - Indigenous Program Manager - Indigenous PM - 1 Indigenous Clinical Nurse Consultant (CNC) - 1 IHW coordinator - 3 Advanced IHW - 2 IHW - 2 Indigenous AH Assistants (AHA) - 1 Indigenous school-based trainee - 2 non-Indigenous CNs |

*This community is serviced by an outreach team because there is no health centre on the island.

** This PHCC was without a permanent PM and had locum CNs for the majority of the implementation phase.
